# Identification of Brain Activation Areas in Response to Active Tactile Stimulation by Gripping a Stress Ball

**DOI:** 10.3390/brainsci15030264

**Published:** 2025-02-28

**Authors:** Kei Sasaki, Noriko Sakurai, Nobukiyo Yoshida, Misuzu Oishi, Satoshi Kasai, Naoki Kodama

**Affiliations:** 1 Department of Radiological Technology, Faculty of Medical Technology, Niigata University of Health and Welfare, 1398 Shimami-cho, Kita-ku, Niigata 950-3198, Japan; kei-sasaki@nuhw.ac.jp (K.S.); noriko-sakurai@nuhw.ac.jp (N.S.); nobukiyo-yoshida@nuhw.ac.jp (N.Y.); satoshi-kasai@nuhw.ac.jp (S.K.); 2 Graduate School of Health and Welfare, Niigata University of Health and Welfare, 1398 Shimami-cho, Kita-ku, Niigata 950-3198, Japan; hxm23002@nuhw.ac.jp

**Keywords:** pleasant tactile perception, active touch, stress ball hardness, fMRI brain activity, comfort and discomfort

## Abstract

Background/Objectives: Research on pleasant tactile perception has primarily focused on C-tactile fibers found in hairy skin, with the forearm and face as common study sites. Recent findings of these fibers in hairless skin, such as the palms, have sparked interest in tactile stimulation on the hands. While studies have examined comfort and brain activity in passive touch, active touch remains underexplored. This study aimed to investigate differences in pleasant sensation and brain activity during active touch with stress balls of varying hardness. Methods: Forty healthy women participated. Using functional magnetic resonance imaging (fMRI), brain activity was measured as participants alternated between gripping stress balls of soft, medium, and hard hardness and resting without a ball. Participants rated hardness and comfort on a 9-point scale. Results: Soft stress balls were perceived as soft and comfortable, activating the thalamus and left insular cortex while reducing activity in the right insular cortex. Medium stress balls elicited similar perceptions and thalamic activation but with reduced right insular cortex activity. Hard stress balls caused discomfort, activating the insular cortex, thalamus, and amygdala while reducing anterior cingulate cortex activity. Conclusions: Soft stress balls may reduce aversive stimuli through perceived comfort, while hard stress balls may induce discomfort and are unlikely to alleviate stress.

## 1. Introduction

The physiological basis for pleasant tactile perception has been investigated by neuroscientists, with particular attention given to C-tactile fibers. These unmyelinated, low-threshold mechanosensory afferents are most strongly activated by slow, gentle stroking, such as petting a dog [1,2,3,4]. C-tactile fibers were previously thought to be present only in hairy skin, such as the forearm and face, but have also recently been shown to be present in hairless skin [5]. A typical example of hairless skin is the palm. The human palm is essential for holding and grasping objects and is central to the tactile function. Nevertheless, most studies using pleasant tactile stimuli have been conducted on hairy skin, such as the forearm, and hairless skin has not been the site of study [6,7]. Since C-tactile fibers have been found in the palm [5], evaluating tactile stimulation on hairless skin is crucial for understanding the brain mechanisms associated with pleasant stimuli.

Neuroanatomical regions related to somatosensory perception have been studied using functional magnetic resonance imaging (fMRI) [8,9]. fMRI utilizes the blood oxygen level-dependent (BOLD) effect. When neuronal activity increases, oxygen consumption in the corresponding brain region rises. In response, blood flow increases, supplying oxygenated hemoglobin. Oxygenated hemoglobin is less susceptible to magnetic fields, whereas deoxygenated hemoglobin is more affected. Generally, increased neuronal activity leads to greater oxygen supply, reducing the proportion of deoxygenated hemoglobin. By detecting these changes, fMRI visualizes activated brain regions [10]. However, MRI may pose challenges such as claustrophobia, psychological stress, noise, and mild invasiveness due to magnetic fields. Therefore, near-infrared spectroscopy (NIRS) is sometimes used as a non-invasive method for measuring brain activity [11]. NIRS employs near-infrared light to measure oxygenated and deoxygenated hemoglobin within tissues, allowing for the real-time monitoring of blood flow. However, NIRS is primarily limited to measuring superficial tissues and cannot directly observe deeper brain structures. Thus, despite its mild invasiveness, MRI is more suitable for observing deep brain regions involved in processing pleasant and unpleasant sensations, such as the insular cortex, anterior cingulate cortex, amygdala, and nucleus accumbens.

Somatosensory perception consists of two systems: the lemniscal system (somatosensory–motor system) and the neospinothalamic system (primarily responsible for pain, temperature, and crude touch). The activation patterns of these regions vary depending on the type of sensory stimulus, experimental task, and stimulus intensity. Observations using fMRI reflect localized blood flow changes in response to stimuli, contributing to the understanding of information processing networks within each sensory system. Furthermore, these systems give rise to both protopathic and epicritic sensations [12]. In particular, epicritic sensation is responsible for detecting fine and precise tactile information, enabling the accurate identification of an object’s shape, texture, and location. While this epicritic sensation specializes in the discrimination of physical stimuli, affective touch mediated by C-tactile fibers is involved in processing pleasant and pleasurable sensations [1,2,3,4]. However, these sensory modalities are not entirely independent and exhibit interactions. For example, the recognition of fine tactile details (epicritic sensation) can influence the evaluation of tactile pleasure. Smooth and soft materials like silk or velvet are perceived as “soft” through discriminative touch, enhancing the sensation of pleasure.

Neuroanatomical studies using fMRI have revealed significant activation in areas such as the anterior cingulate cortex, orbitofrontal cortex, and amygdala during pleasant passive touch on hairless skin [13]. In addition, Francis et al. found significant activation in the primary somatosensory cortex (S1), orbitofrontal cortex, and thalamus when pleasant stimulation was applied to the palm [14]. The insular cortex and thalamus are responsible for controlling emotions such as pleasant and unpleasant feelings [15,16]. Furthermore, sex differences in tactile sensitivity have been reported, with women showing greater sensitivity and higher comfort ratings for emotional tactile stimuli [17].

Although textured methods of providing pleasant stimulation, including brushes and cloths, have received more attention, other tactile stimuli, such as deep pressure, remain underexplored. Deep pressure is one of the pleasant tactile sensations that can be easily studied in the laboratory. It is experienced during activities like hugging, holding objects, or holding infants; is a fundamental tactile sensation; and is incorporated into many manual therapies, such as massage therapy. Massage therapy has been shown to significantly reduce depression, stress, and pain, as well as improve immune response in adults and promote weight gain in infants [18,19]. In addition, occupational therapists practice deep pressure touch, which reduces anxiety and increases calmness [20,21]. Moderate pressure has similar benefits, reducing stress and anxiety while improving comfort ratings [22,23]. Although interpersonal contact is implicated in the results of these studies, Laura et al. demonstrated that specific patterns of deep pressure remain pleasant when applied mechanically, even in the absence of social interaction [24]. However, these studies primarily evaluated the effects of passive touch, where tactile stimulation is provided by an experimenter. The impact of active touch, where participants generate tactile stimulation through their touch or grip, remains unclear.

This study used a stress ball to investigate tactile perception and brain activity with tactile stimulation in active touch. Stress balls are small, inexpensive, and portable with various types made from polyurethane foam rubber or gel. Clinically, stress balls are used for physical therapy, during endoscopy [25], and to reduce anxiety and pain in patients undergoing hemodialysis [26]. However, previous studies only evaluated the effects of stress balls on humans subjectively through questionnaires, leaving the influence of active touch on brain activity unexplored. The tactile perception of hardness reveals that soft objects are generally perceived as more comfortable than hard ones [13,14,27]. Pleasant tactile stimulation offers emotional and psychological support that helps reduce social isolation and stress [28]. Conversely, hard tactile stimulation to the fingertips can cause discomfort [29].

The fingertips and palms of human hands are essential for actively grasping and touching objects in daily life. Despite this, most previous studies using fMRI have employed passive stimulation [24,30,31], and it remains unclear how differences in object hardness are processed in the human brain during active tactile stimulation. One possible reason for this gap is the difficulty in ensuring experimental reproducibility. In previous studies using passive stimulation, disk- or drum-type skin stimulation devices, which allow for controlled rotation speed and stimulation duration, have been used [32]. This approach ensures reproducibility and facilitates experimentation. In contrast, studies involving active stimulation require participants to control the force and speed at which they touch or grasp objects. Additionally, since magnetic materials cannot be used inside an MRI scanner, it is challenging to regulate gripping force and pace mechanically. To address this issue, Sasaki et al. successfully conducted an fMRI experiment using active stimulation by training participants with a handgrip dynamometer and a metronome [27]. Although the control of active exploration processes during experiments is challenging, these processes play a crucial role in perceiving object hardness. Furthermore, active tactile stimulation has been shown to enhance the discrimination of an object’s tactile properties more effectively than passive stimulation [33]. While passive stimulation has been associated with soft textures inducing pleasant sensations and corresponding brain activations [13,14], it remains unclear how differences in hardness affect pleasant sensations and brain activation patterns during active stimulation. Thus, identifying brain activation differences related to variations in object hardness during active stimulation and evaluating subjective pleasure could be a crucial step toward understanding the neural mechanisms underlying pleasure processing in active touch. Moreover, stress balls used in this study can be utilized in various situations, such as during studying, daily activities, and even during medical procedures like surgery or examinations [25,26]. Compared to passive stimulation methods, such as massage, which require a third party, stress balls offer a more accessible and practical approach to mental healthcare.

In passive tactile stimulation, softer stimuli tend to induce greater pleasure, with significant activation observed in the anterior cingulate cortex, orbitofrontal cortex, amygdala, primary somatosensory cortex (S1), orbitofrontal cortex, and thalamus [13,14]. However, in fMRI studies using active tactile stimulation, the brain activation regions associated with differences in object hardness remain unclear. Furthermore, the relationship between hardness and pleasure during active stimulation has not been fully elucidated. Therefore, this study aimed to identify brain activation areas and differences in perceived pleasure in response to varying object hardness during the active squeezing of a stress ball using fMRI. By clarifying the neural correlates and subjective pleasure differences in active stimulation with a stress ball, this research could contribute to a deeper understanding of the fundamental neural mechanisms underlying pleasant touch and other forms of active tactile interactions. Additionally, it may help propose emotional and therapeutic benefits associated with active tactile stimulation.

## 2. Materials and Methods

### 2.1. Participants

The participants were 40 healthy women (mean age 21.7 ± 1.4 years) over the age of 20 who had not previously undergone an fMRI examination while holding a stress ball. All participants were right-handed and had no history of mental disorders or medication use. Previous studies have shown that there are sex differences in somatosensory sensitivity, with women being more sensitive to touch than men and reporting higher ratings of pleasantness in response to affective tactile stimulation [17]. Therefore, this study considered these differences in sensitivity and recruited only female participants. Additionally, since psychiatric disorders such as autism spectrum disorder and depression may affect tactile sensitivity [34,35], only healthy individuals were included in the study.

This study was approved by the Ethical Review Committee of Niigata University of Health and Welfare (approval number: 18992-230203). All researchers complied with the Declaration of Helsinki. Informed consent was obtained from all participants, who were asked to complete a medical questionnaire to ensure their safety during MRI imaging.

### 2.2. Stimuli Task

**Figure 1:**
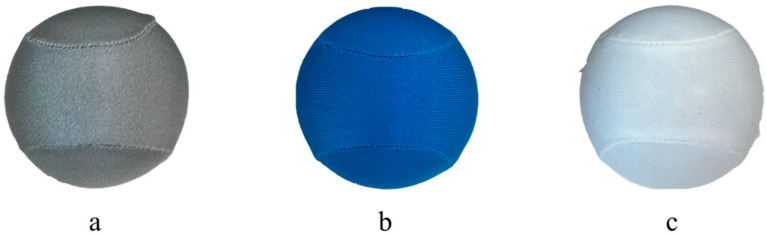
Stress ball held by the participant: (**a**) soft; (**b**) medium; (**c**) hard.

The stress balls (Serenilite, Great Neck, NY, USA) used in this study are shown in Figure 1. Three levels of hardness were tested: soft, medium, and hard. Each stress ball consisted of a smooth Lycra fabric surface and a high-resistance interior. Hardness was measured using a Qiilu digital durometer (type A), showing 0.5 HA for soft, 2.5 HA for medium, and 5.5 HA for hard. Participants were instructed to squeeze each stress ball with a force of 5 kg once per second. To prepare for the task, participants used a grip strength meter to learn the required 5 kg force prior to entering the MRI room. A metronome was also used to help them remember the pace of one grip per second. To eliminate visual bias, the stress ball was not shown to the participants beforehand, and their eyes remained closed during the MRI imaging.

### 2.3. Block Design

**Figure 2:**
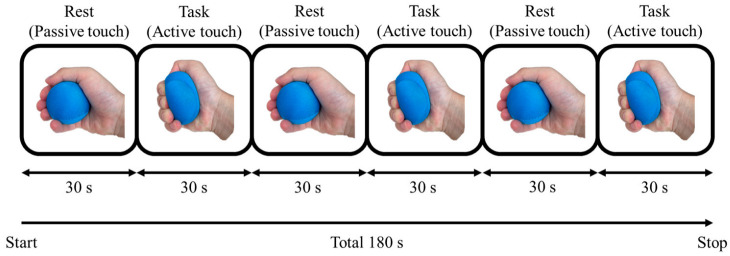
Block design. Rest (passive touch) was repeated three times for 30 s, and task (active touch) was repeated three times for 30 s, for a total of 3 min.

The block design of this study is shown in Figure 2. In passive touch, no exercise was performed, and the stress ball was held in the hand; in active touch, the stress ball was held once per second with a force of 5 kg. Passive touch was performed for 30 s and served as a rest period, while each active touch was performed for 30 s and served as the task period, alternating three times for a total of 3 min. Since there were three different stress balls, the block design was imaged three times while replacing the stress ball. Additionally, we analyzed changes in BOLD activation using this block design for active touch and identified the brain regions activated in response to tactile stimulation.

### 2.4. Apparatus

Imaging was performed using a 3-tesla MRI system (Vantage Galan, Canon Medical Systems, Tochigi, Japan) with a 32-channel head coil. Participants lay supine on the MRI machine bed with their arms extended along their bodies and palms facing upward. A sponge was placed between the head coil and the participant’s head to minimize head movement during the task. The stress ball was placed in the palm of the participant’s right hand and replaced by the experimental assistant after each block. The order of stress ball presentation was randomized to eliminate any order effects.

### 2.5. MRI Acquisition

High-resolution MRI images were captured separately to obtain detailed anatomical information before fMRI imaging. A high-resolution T1-weighted magnetization-prepared rapid gradient-echo (MP-RAGE) sequence was used for structural imaging. The settings were repetition time (TR) = 5.8 ms, echo time (TE) = 2.7 ms, inversion time (TI) = 900 ms, flip angle (FA) = 9°, number of matrix (matrix) = 256 × 256, field of view (FOV) = 230 × 230 mm, and slice thickness = 1.2 mm. Echo-planar imaging (EPI) sequences were used to capture fMRI images with the following parameters: TR = 2000 ms, TE = 25 ms, FA = 85°, matrix = 64 × 64, FOV = 240 × 240 mm, and slice thickness = 3 mm to ensure complete brain coverage.

### 2.6. fMRI Data Analyses

The fMRI data were preprocessed and analyzed using Statistical Parametric Mapping 12 (SPM12; Wellcome Trust Center for Neuroimaging) in Matlab (Mathworks Inc., Natick, MA, USA). Slice timing correction was used to adjust for time differences. Subsequently, realignment parameters were used to correct for motion-related displacement. In addition, we used co-registration to compare structural and fMRI images, correcting for any misalignment. Normalized images were created by mapping each participant’s brain to the Montreal Neurological Institute (MNI) standard brain coordinate system. The normalized images were smoothed using an 8 mm Gaussian kernel. After preprocessing, a block design using the general linear model was used to identify changes in brain activity related to stimulation by active touch for each stimulus task. Contrast images were created at (1) soft = 1, medium = 0, hard = 0; (2) soft = 0, medium = 1, hard = 0; and (3) soft = 0, medium = 0, hard = 1 at the first level (individual analysis). We also identified brain regions that were less active due to the task by taking the difference between brain-activated regions in rest (passive touch) and brain-activated regions in the task (active touch). The contrasts were created at (4) soft = −1, medium = 0, hard = 0; (5) soft = 0, medium = −1, hard = 0; and (6) soft = 0, medium = 0, hard = −1. In addition, specific brain activation sites for each stress ball were investigated, with contrasts created at (7) soft = 1, medium = −1, hard = 0; (8) soft = −1, medium = 1, hard = 0; (9) soft = 1, medium = 0, hard = −1; (10) soft = −1, medium = 0, hard = 1; (11) soft = 0, medium = 1, hard = −1; and (12) soft = 0, medium = −1, hard = 1. At the second level (population analysis), a one-sample *t*-test was performed using the above contrasts. The initial threshold for voxel level was set at uncorrected *p* < 0.001. Cluster size was considered significant if *p* < 0.05, corrected for family-wise error (FWE) due to peak level.

### 2.7. Questionnaire

After fMRI imaging, participants completed a Likert scale-based questionnaire. They rated the “softness/hardness” and “comfort/discomfort” of each stress ball on a 9-point scale. SPSS (IBM SPSS Statistics Base) 27.0 was used for statistical analysis. The Kruskal–Wallis test was used for the hardness and comfort ratings, and multiple comparisons with Bonferroni correction were performed after testing for differences between groups. The correlation between comfort scores and clusters significant by fMRI was also investigated. Both tests used a significance threshold of 5%.

## 3. Results

### 3.1. Brain Activation Sites When Holding a Stress Ball

**Figure 3:**
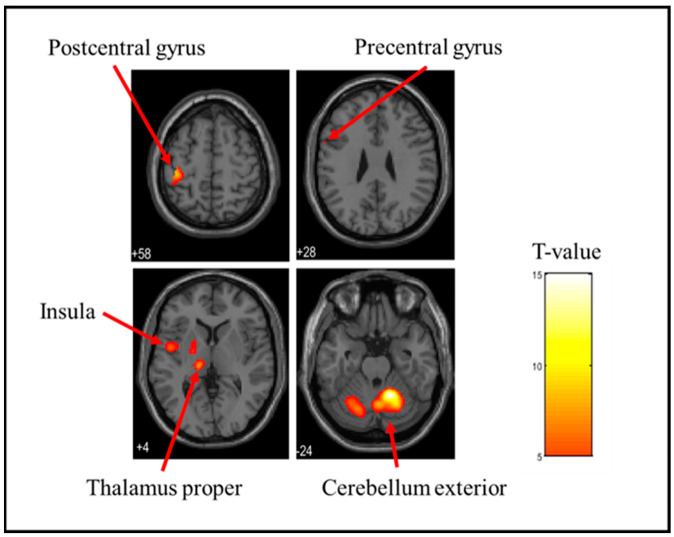
Brain activation during stress ball (soft) grip. The figure shows the activation of the postcentral gyrus, precentral gyrus, insula, thalamus proper, and cerebellum exterior. The T-value indicates the intensity of activation.

**Figure 4:**
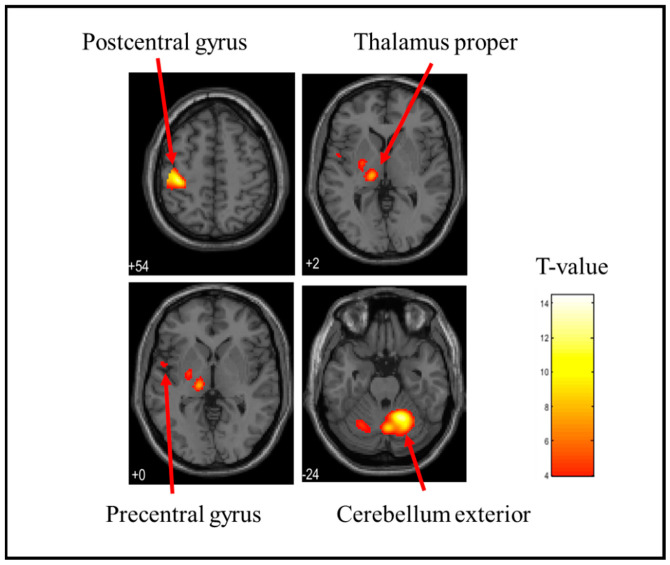
Brain activation during stress ball (medium) grip. The figure shows the activation of the postcentral gyrus, precentral gyrus, thalamus proper, and cerebellum exterior. The T-value indicates the intensity of activation.

**Figure 5:**
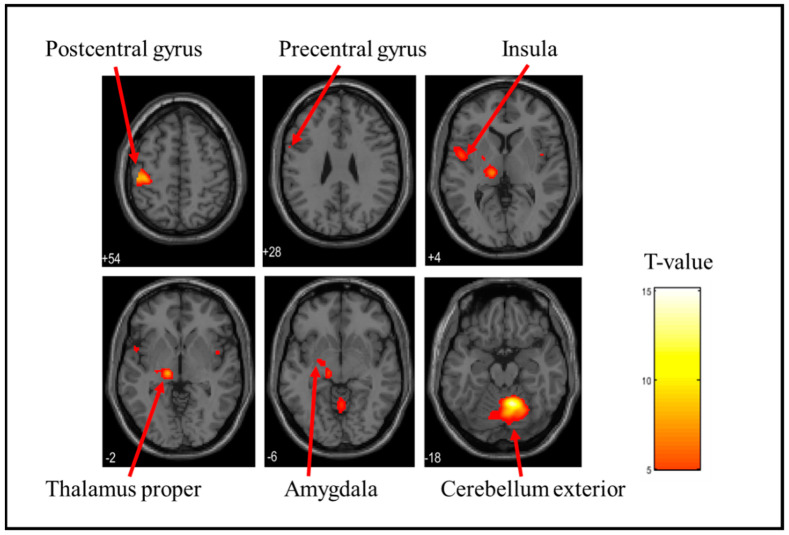
Brain activation during stress ball (hard) grip. The figure shows the activation of the postcentral gyrus, precentral gyrus, insula, thalamus proper, amygdala, and cerebellum exterior. The T-value indicates the intensity of the activation.

In this study, fMRI was used to identify brain regions exhibiting BOLD activation. BOLD activation refers to the phenomenon in which changes in blood oxygen levels associated with neural activity are detected as MRI signals. By analyzing these changes in BOLD activation, we identified the brain regions activated in response to tactile stimulation. Table 1 shows the brain activation sites when each stress ball was held. The cerebellum exterior, postcentral gyrus, thalamus proper, anterior insula, parietal operculum, putamen, and precentral gyrus were considerably activated when the soft stress ball was held. The postcentral gyrus, cerebellum exterior, thalamus proper, parietal operculum, caudate, putamen, and precentral gyrus were considerably activated when holding a medium stress ball. During the hard stress ball grip, the cerebellum exterior, postcentral gyrus, thalamus proper, anterior insula, parietal operculum, middle frontal gyrus, central operculum, amygdala, precentral gyrus, opercular part of the inferior frontal gyrus, and supramarginal gyrus were markedly activated. However, the specific brain activation sites for each of the three types of stress balls were investigated by differential contrast imaging of all six patterns, and no significant activation was observed in any of the patterns. Figure 3, Figure 4 and Figure 5 also show the brain activation when gripping soft, medium, and hard stress balls, respectively.

### 3.2. Brain Regions with Reduced Activity During Stress Ball Gripping

**Figure 6:**
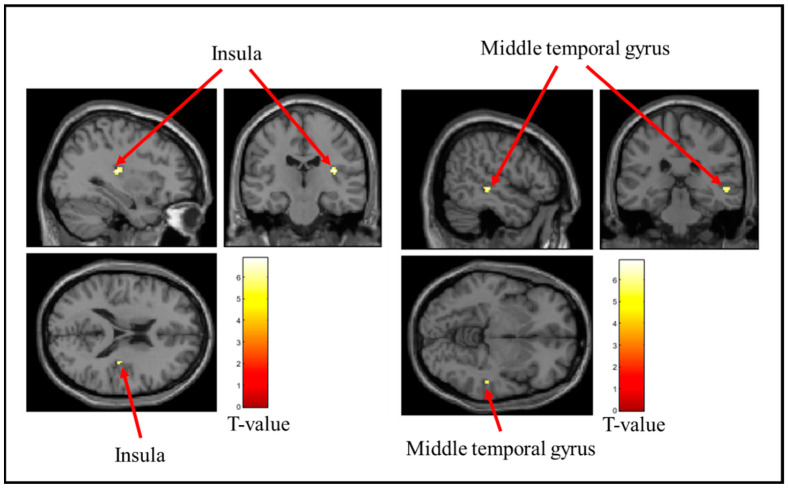
Brain regions that were significantly less active when the soft stress ball was gripped. The figure shows the insula and middle temporal gyrus, which were significantly less active when the soft stress ball was grasped. The T-value indicates the intensity of the decrease in activity.

**Figure 7:**
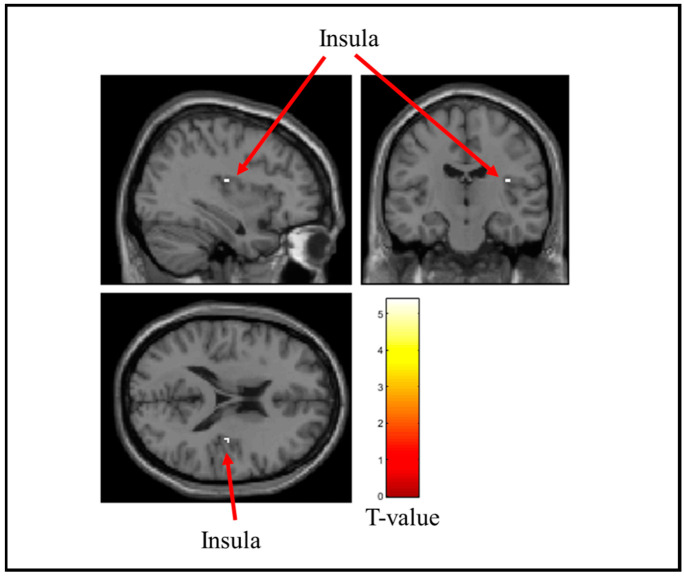
Brain regions that were significantly less active when the medium stress ball was gripped. The figure shows the insula, which showed a significant decrease in activity when the medium stress ball was gripped. The T-value indicates the intensity of the decrease in activity.

**Figure 8:**
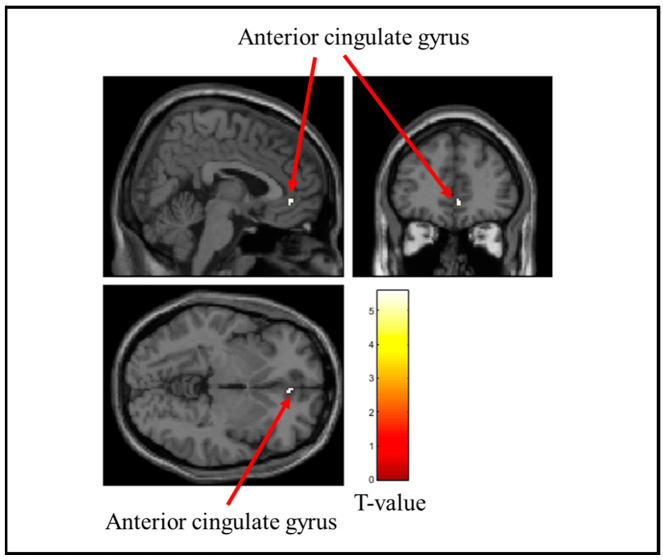
Brain regions that were significantly less active when gripping the hard stress ball. The figure shows the anterior cingulate gyrus, which was significantly less active during the hard stress ball grip. The T-value indicates the intensity of the decrease in activity.

Table 2 lists the brain regions that showed decreased activity when the stress ball was grasped. Figure 6, Figure 7 and Figure 8 show the brain regions that were remarkably less active when the soft, medium, and hard stress balls were held, respectively. Activity in the insular cortex and middle temporal gyrus was markedly reduced during the gripping of the soft stress ball. For the medium stress ball, activity in the insular cortex was also considerably reduced. Additionally, activity in the anterior cingulate gyrus was considerably reduced when the hard stress ball was gripped.

### 3.3. Results of Survey Score Analysis

The results of the questionnaire assessing the comfort of gripping the stress ball are shown in Table 3. The mean score for comfort when holding the soft stress ball was 6.7 ± 3.7, for the medium stress ball 5.9 ± 3.9, and for the hard stress ball 4.6 ± 3.1. The Kruskal–Wallis test revealed a significant difference (χ^2^ = 24.4, *p* < 0.001). Therefore, multiple comparisons were made to compare the comfort scores of each stress ball, and significant differences were found between soft and medium (*p* = 0.045), soft and hard (*p* < 0.001), and medium and hard (*p* = 0.0040). Furthermore, no correlations were found between comfort scores and brain activation patterns.

The results of the questionnaire assessing the hardness of the stress ball when gripped are shown in Table 4. The mean hardness score for gripping the soft stress ball was 3.1 ± 5.0, for the medium stress ball 5.2 ± 5.0, and for the hard stress ball 7.1 ± 4.8. The Kruskal–Wallis test indicated a significant difference (χ^2^ = 64.7, *p* < 0.001). Therefore, multiple comparisons were conducted to compare the hardness scores of each stress ball, and significant differences were found between soft and medium (*p* < 0.001), soft and hard (*p* < 0.001), and medium and hard (*p* < 0.001). Furthermore, no correlations were found between hardness scores and brain activation patterns.

## 4. Discussion

This study aimed to examine and clarify the differences in comfort evaluation and brain activation sites when using stress balls of varying hardness. Remarkable activation was observed in brain regions related to emotion regulation, such as the insular cortex, hypothalamus, and amygdala when participants held stress balls with three different hardness levels. We also identified brain regions that were markedly less active when the stress ball was gripped. Specifically, activity in the insular cortex and middle temporal gyrus was considerably reduced when gripping the soft stress ball; activity in the insular cortex was reduced when gripping the medium stress ball; and activity in the anterior cingulate cortex was markedly reduced when gripping the hard stress ball. The questionnaire results confirmed that participants could distinguish between the three stress ball hardness levels. They rated the soft and medium stress balls as comfortable to grip, while the hard stress ball was perceived as uncomfortable. Since no significant correlation was observed between the scores and the significant clusters of brain activation, it remains unclear whether the pleasant sensation induced by active stimulation activated each brain region.

In common with stress balls of all hardness levels, notable activation of the postcentral gyrus, precentral gyrus, and cerebellum exterior was found. The postcentral gyrus is located in the somatosensory cortex, which processes information obtained from the sensory receptors. The primary motor cortex is located in the precentral gyrus, which collaborates with the premotor cortex in planning and executing movements. The cerebellum is functionally connected to a sensory–motor network that combines the sensory–motor cortex, premotor cortex, and supplementary motor cortex and is involved in the control, learning, and planning of movement [36,37,38,39,40]. The cerebellum has also been reported to be activated during the perception of hardness in tactile functions [41]. Furthermore, in our previous study, significant activation of these regions was commonly observed when gripping objects of various textures [27]. The present experimental data support that the postcentral gyrus, precentral gyrus, and cerebellum exterior are markedly activated by active tactile stimulation when the stress ball is grasped.

Strong activation was found in the thalamus and insular cortex with respect to the soft stress ball. The thalamus and insular cortex are responsible for controlling emotions and have been reported to be activated by both pleasant and unpleasant emotions [15,16]. Therefore, since the activated brain regions alone could not be used to determine pleasant and unpleasant feelings, we identified brain regions whose activity was reduced by grasping the stress ball. The results revealed that activity in the insular cortex was considerably reduced. The insular cortex tends to increase blood flow under physical or mental stress. Conversely, training in mindfulness, which has stress-reducing and relaxing effects, markedly reduces activation of the right insular cortex during aversive breath-loading tasks [42]. It has also been shown that comfort is produced by soft tactile stimuli [13,14,27]. The results of the questionnaire scores showed that the soft stress ball had a hardness score of 3.1 ± 5.0 and a comfort score of 6.7 ± 3.7. This indicates that it was considerably softer and more comfortable than the other hard stress balls. Thus, it was suggested that gripping the soft stress ball produced comfort and reduced aversive or unpleasant stimuli, similar to the effects produced by mindfulness.

Strong activation of the thalamus was observed in the medium stress ball. The thalamus plays a crucial role in processing sensory information, transmitting tactile information from the ventral posterior medial nucleus to the primary somatosensory cortex (S1) for detailed tactile processing. This thalamocortical interaction enables the recognition of an object’s shape, texture, and position through touch [43]. It is closely related to the amygdala, which influences homeostatic mechanisms and neuroendocrine signaling [44]. However, as with the soft stress ball, activated brain regions alone cannot determine pleasure or discomfort. Therefore, we identified the regions of the brain that were markedly less activated by gripping. The results showed a considerable decrease in activity in the right insular cortex similar to the findings with the soft stress ball. Questionnaire scores for the medium stress ball were 5.2 ± 5.0 for hardness and 5.9 ± 3.9 for comfort, suggesting it was perceived as softer and more comfortable than neutral but less so than the soft stress ball. These findings indicate that gripping the medium stress ball is likely to reduce aversive or unpleasant stimuli, comparable to the effects associated with mindfulness [42].

The hard stress ball showed marked activation in the insular cortex, thalamus, and amygdala. The insular cortex and thalamus, as previously noted, are responsible for processing both pleasant and unpleasant emotions. The amygdala is also known for its role in emotional processing, particularly in generating negative emotions such as fear, as demonstrated in animal and human studies [45,46]. However, several amygdala functions have also been reported for positive emotions such as pleasure. For example, it is involved in brain circuits associated with happiness and pleasant sensations [47,48,49]. Thus, the amygdala has been shown to be involved in the processing of both pleasant and unpleasant emotions, but it is not possible to tell which of the pleasant or unpleasant emotions is being generated by the activated brain region alone. Notably, activity in the anterior cingulate cortex was markedly reduced when the hard stress ball was held. The anterior cingulate cortex is the region responsible for behavior monitoring, social cognition, and emotion. In particular, it has been reported that blood flow in the anterior cingulate cortex is reduced by the presentation of images that elicit unpleasant emotions [50]. The anterior cingulate cortex also has strong connectivity with the amygdala [51] and is thought to play an important role in emotion processing [52]. In addition, the questionnaire results for gripping a hard stress ball showed a hardness score of 7.1 ± 4.8 and a comfort score of 4.6 ± 3.1. This indicates that gripping the hard stress ball caused perceived hardness and discomfort. Furthermore, previous studies have reported that hard tactile stimuli can cause discomfort [29]. This evidence suggests that gripping a hard object in the present study produced discomfort, suggesting that the hard stress ball is not expected to be effective in reducing stress or unpleasant stimuli.

There are some limitations to this study: the first is the examination of sex differences. In this study, only women were included as participants, and the gripping strength for the stress ball was set at 5 kg. The results indicated a preference for soft and medium levels, while hard levels were associated with discomfort. However, it is believed that preferences for the hardness of stress balls and the comfort level depending on grip strength may differ by sex. Therefore, it is necessary to conduct experiments with men as well and perform a comparative analysis. Second, this study did not target individuals with high anxiety or stress levels. Comfort is expected to alleviate anxiety and stress. Therefore, it is necessary to investigate whether the use of questionnaires such as the STAI (State-Trait Anxiety Inventory) and SRS-18 (Stress Response Scale) can demonstrate a reduction in anxiety and stress in individuals with high anxiety or high-stress levels. Additionally, the potential clinical applications of stress balls, including their use in treatment and pain relief, warrant further exploration. Third, ensuring reproducibility is essential. In this study, the grip strength when squeezing the stress ball was trained using a handgrip dynamometer, but there was no control during the MRI scanning. However, due to the restriction of bringing magnetic objects such as the dynamometer into the MRI room, it is necessary to develop new stimulation tasks and monitoring methods during scanning to eliminate this concern. Additionally, as each stress ball was only scanned once in this study, it was difficult to distinguish between the effects of the type of stress ball and the impact of MRI scanning. Therefore, performing multiple scans for a single task is expected to improve statistical reliability and reduce individual differences and the effects of MRI scanning. Fourth, the direct pattern comparison between different tasks is crucial. In this study, we conducted contrast analyses to directly compare brain activation patterns between different stress balls. However, no significant activation was observed in any of the contrasts. This result may be attributed to factors such as individual differences, limitations in sample size, or the possibility that the neural representation of hardness/softness is distributed rather than localized.

## 5. Conclusions

This study aimed to evaluate the comfort associated with differences in the hardness of stress balls and investigate differences in brain activation regions. The participants rated the soft and medium stress balls as comfortable and the hard stress balls as uncomfortable. Brain activity analysis revealed remarkable activation in brain regions involved in emotional regulation, such as the insular cortex, hypothalamus, and amygdala, when gripping the stress balls. Additionally, activity in the right insular cortex decreased when gripping the soft and medium stress balls, whereas activity in the anterior cingulate cortex decreased when gripping the hard stress ball. The questionnaire scores suggested that softer stress balls induced pleasant sensations, while using harder ones seemed to cause discomfort. However, since no significant correlation was observed with the activated clusters, it remains unclear whether the pleasant or unpleasant feelings specifically activated each brain region.

## Figures and Tables

**Table 1:** Brain activation regions when gripping each stress ball.

	Hemisphere	Locations	Cluster *p*-Value (FWE)	Cluster Size (Voxels)	T-Value	X [mm]	Y [mm]	Z [mm]
Soft	Right	Cerebellum Exterior	<0.001	2378	14.98	16	−54	−24
	Left	Postcentral gyrus	<0.001	420	9.88	−40	−26	58
	Left	Thalamus Proper	<0.001	339	8.88	−14	−22	4
	Left	Anterior insula	<0.001	219	7.73	−48	0	2
	Left	Parietal operculum	<0.001	166	7.54	−50	−24	16
	Left	Putamen	<0.001	128	6.95	−22	−6	2
	Left	Precentral gyrus	0.028	3	5.84	−58	8	28
Medium	Left	Postcentral gyrus	<0.001	659	14.41	−38	−26	58
	Right	Cerebellum Exterior	<0.001	1713	13.14	18	−54	−24
	Left	Thalamus Proper	<0.001	515	9.47	−14	−22	4
	Left	Parietal operculum	<0.001	189	9.09	−48	−24	14
	Left	Cerebellum Exterior	<0.001	206	8.73	−28	−62	−28
	Right	Caudate	<0.001	65	7.59	16	−16	20
	Left	Putamen	<0.001	72	7.10	−24	−8	2
	Left	Precentral gyrus	0.012	12	5.47	−52	2	0
Hard	Right	Cerebellum Exterior	<0.001	2479	15.09	18	−56	−22
	Left	Postcentral gyrus	<0.001	454	10.12	−40	−26	56
	Left	Thalamus Proper	<0.001	363	9.66	−14	−20	−2
	Left	Anterior insula	<0.001	275	7.33	−48	0	4
	Right	parietal operculum	<0.001	173	7.07	66	−24	18
	Right	Middle frontal gyrus	0.003	39	6.78	54	4	40
	Left	Central operculum	<0.001	143	6.53	−52	−22	16
	Left	Amygdala	0.005	28	6.00	−22	−10	−4
	Right	Anterior insula	0.001	51	5.73	48	2	10
	Left	Precentral gyrus	0.039	1	5.34	−58	6	28
	Right	Opercular part of the inferior frontal gyrus	0.039	1	5.23	60	10	16
	Right	Supramarginal gyrus	0.039	1	5.23	60	−26	46

“Hemisphere” indicates which hemisphere (left or right) showed activation. “Locations” lists the names of the significantly activated brain regions. “Cluster *p*-value (FWE)” represents the *p*-value corrected using FWE. “Cluster size (voxels)” indicates the extent of the significant cluster in terms of the number of voxels. “T-value” represents the T-value of significant activation after FWE correction. “X, Y, Z [mm]” provides the three-dimensional coordinates used to indicate spatial locations in MRI and fMRI images. The X-coordinate represents lateral positioning, with positive values indicating the right hemisphere and negative values indicating the left hemisphere. The Y-coordinate represents anterior-posterior positioning, with positive values indicating the frontal region and negative values indicating the occipital region. The Z-coordinate represents vertical positioning, with positive values indicating superior regions and negative values indicating inferior regions.

**Table 2:** Brain regions with decreased activity when holding a stress ball.

	Hemisphere	Locations	Cluster *p*-Value (FWE)	Cluster Size(Voxels)	T-Value	X [mm]	Y [mm]	Z [mm]
Soft	Right	Insula	0.001	35	6.88	34	−20	16
	Right	Middle temporal gyrus	0.006	23	6.09	52	−28	4
Medium	Right	Insula	0.034	3	5.39	36	−18	18
Hard	Right	Anterior cingulate gyrus	0.02	8	5.57	4	42	−4

**Table 3:** Comparison of comfort scores when holding each stress ball.

Likert Scale Point	Unpleasant								Pleasant	Ave
	1	2	3	4	5	6	7	8	9	
Soft	1	0	1	4	2	7	10	9	6	6.7 ± 3.7
Medium	0	1	4	3	5	11	10	5	1	5.9 ± 3.9
Hard	2	4	8	6	5	10	2	2	1	4.6 ± 3.1

Higher score numbers indicate more comfort; lower scores indicate discomfort. A score of 5 indicates neither.

**Table 4:** Comparison of hardness scores when gripping each stress ball.

Likert Scale Point	Soft								Hard	Ave
	1	2	3	4	5	6	7	8	9	
Soft	6	8	16	3	3	1	2	1	0	3.1 ± 5.0
Medium	0	0	2	12	9	11	5	1	0	5.2 ± 5.0
Hard	0	1	1	1	0	9	10	12	6	7.1 ± 4.8

A higher score number indicates that the ball is judged harder, and a lower score indicates that the ball is judged softer. A score of 5 indicates neither.

## Data Availability

The data used in this study will be made available upon reasonable request to the corresponding author. The data are not publicly available as they contain information that could compromise the privacy of research participants.
